# Supplementary material for: Cell type-specific modulation of sensory and affective components of itch in the periaqueductal gray
Source: Nat Commun. 2019 Sep 25;10:4356. doi: 10.1038/s41467-019-12316-0 (PMC6761157; doi:10.1038/s41467-019-12316-0)
Supplement: Supplementary file 1 — Supplementary Information [file 41467_2019_12316_MOESM1_ESM.pdf]

Supplementary Information

Samineni et al., **Cell type-specific modulation of sensory and affective components of itch in the periaqueductal gray**

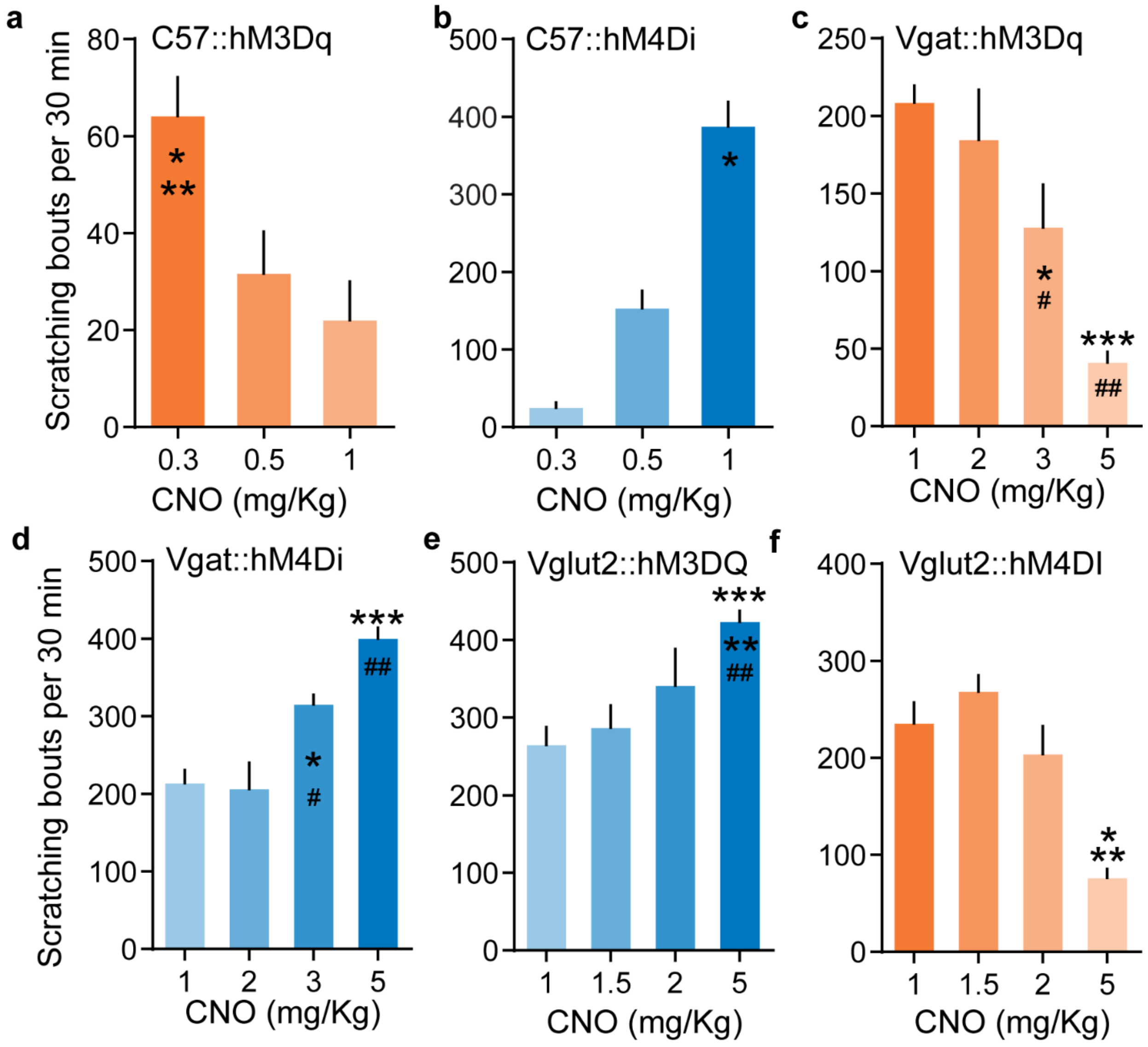

Supplementary Figure 1. CNO administration showed dose dependent effects in mice injected with Cre independent and Cre dependent DREADDs.

(A) CNO administration produced dose dependent decrease in chloroquine evoked scratching in hM3Dq injected mice (one-way ANOVA, 0.3 vs 1 mg/Kg, * P = 0.0144, *N* = 6 for 0.3 mg/kg, *N* = 6 for 0.5 mg/kg and *N* = 15 for 1 mg/kg). (B) CNO administration produced dose dependent increase in chloroquine evoked scratching in hM4Di injected mice (one-way ANOVA, 0.3 vs 1 mg/Kg, **P <0.0001, 0.5 Vs 1, *P = 0.0003; *N* = 6 for 0.3 mg/kg, *N* = 6 for 0.5 mg/kg and *N* = 14 for 1 mg/kg). (C) CNO activation of vlPAG Vgat::hM3Dq neurons produced dose dependent

decrease in chloroquine evoked scratching (one-way ANOVA, *1 vs 3 mg/Kg, P = 0.034, ***1 vs 5, P <0.0001, #2 vs 3, P = 0.033, ##2 vs 5, P <0.0001; *N* = 8 for 1 mg/kg, *N* = 9 for 2 mg/kg, *N* = 6 for 3 mg/kg and *N* = 12 for 5 mg/kg). (D) CNO activation of vlPAG Vgat::hM4Di neurons produced dose dependent increase in chloroquine evoked scratching (one-way ANOVA, *1 vs 3 mg/Kg, P = 0.021, ***1 vs 5, P <0.0001, #2 vs 3, P = 0.0082, ##2 vs 5, P <0.0001; *N* = 8 for 1 mg/kg, *N* = 10 for 2 mg/kg, *N* = 9 for 3 mg/kg and *N* = 8 for 5 mg/kg). (E) CNO activation of vlPAG Vglut2::hM3Dq neurons produced dose dependent increase in chloroquine evoked scratching (one-way ANOVA, 1 vs 5 mg/Kg, **P = 0.0059, 1.5 vs 5, *P = 0.0210; *N* = 7 for 1 mg/kg, *N* = 7 for 1.5 mg/kg, *N* = 10 for 2 mg/kg and *N* = 12 for 5 mg/kg). (F) CNO activation of vlPAG Vglut2::hM4Di neurons produced dose dependent decrease in chloroquine evoked scratching (one-way ANOVA, 1 vs 5 mg/Kg, **P = 0.0005, 1.5 vs 5, ***P <0.0001, 2 vs 5, ##P = 0.0011; *N* = 8 for 1 mg/kg, *N* = 6 for 1.5 mg/kg, *N* = 14 for 2 mg/kg and *N* = 13 for 5 mg/kg). All values are mean ± SEM.

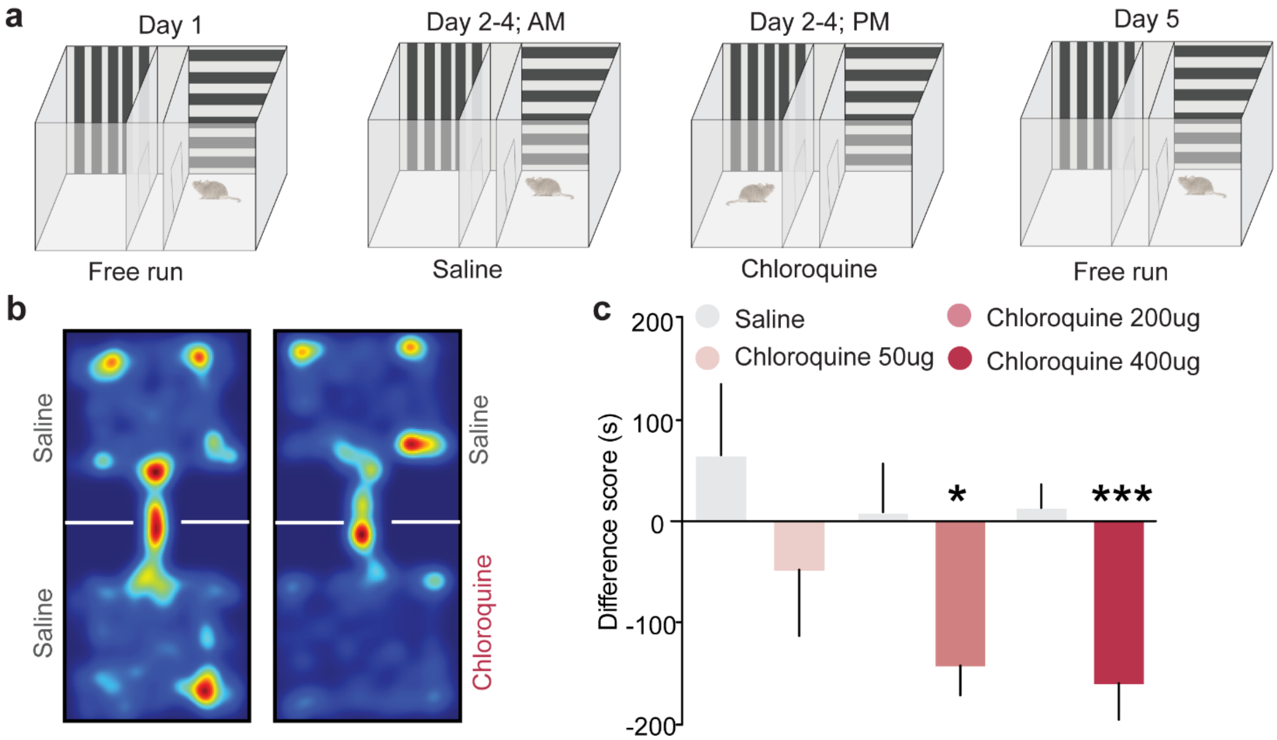

Supplementary Figure 2. Dose dependent chloroquine-induced conditioned place aversion.

(A) Schematic of conditioned place aversion experimental design indicating saline- and chloroquine-paired chambers and the timing of each session. (B) Representative heat maps showing spatial location of a control mouse, pre and post chloroquine conditioning. (C) Upon conditioning mice for 3 days with saline/chloroquine treatment, difference scores indicate that mice spent less time in the chloroquine-paired chamber compared to the saline-paired chamber in a dose dependent manner. A dose of 50ug/50ul chloroquine injection into the nape of the neck did not produce any avoidance to saline or chloroquine chamber (N=5, t-test, t=0.1258, df=8, P=0.7864). While injecting dose of 200ug/50ul (N=6, t-test, t=2.785, df=10, P=0.0193) and 400ug/50ul (N=16, t-test, t=0.1258, df=8, P=0.0002) chloroquine into the nape of the neck produced avoidance to the chloroquine paired chamber. * P=0.0193, *** P=0.0002. All values are mean ± SEM.

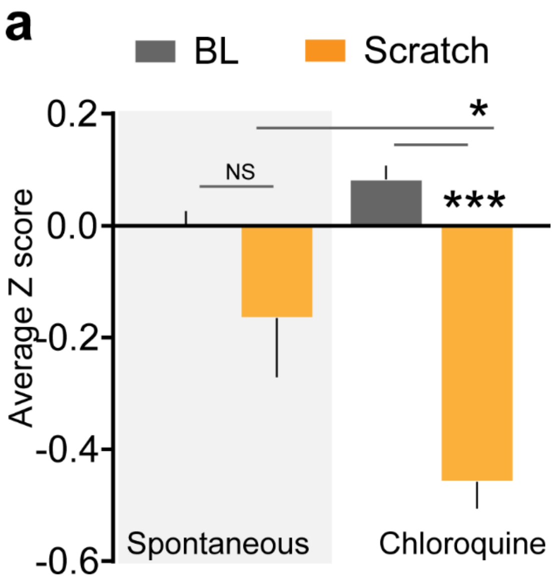

Supplementary Figure 3. Calcium dynamics of vlPAG Vgat+ neurons to spontaneous and chloroquine evoked scratching.

(a) Quantification of Averaged Z score showing no significant differences in the Vgat Ca2+ dynamics between baseline and post spontaneous scratching bout. There is significant decrease in the Vgat Ca2+ activity when compared between spontaneous scratching and chloroquine evoked scratching, suggesting that the decreased activity of these neurons is critical during itch transmission in the PAG. (30 s pre- and post- scratch; *t=2.147, df=4, *P=0.039, t test, t=10.07, df=4, ***P <0.0001)*. All values are mean ± SEM.

Supplementary Discussion

There were some notable discrepancies between our behavioral data obtained from pharmacological and chemogenetic manipulation of global PAG activity compared to cell-type specific PAG manipulations. First, global vlPAG inactivation by lidocaine infusion attenuated scratching behaviors, an effect that was not mimicked by global chemogenetic inhibition of vlPAG activity. In fact, global chemogenetic inhibition in the vlPAG enhanced scratching behavior, whereas chemogenetic activation of vlPAG suppressed scratching. This distinction can be explained by either a potential impact of lidocaine on axons of passage in the PAG, or by a predominant or epistatic effect of lidocaine on a specific cell type. Consistent with the latter concept, cell-type-specific chemogenetic inhibition of Vgat and Vglut2 neurons had opposing actions, where inhibition of Vglut neurons caused suppression of scratching and inhibition of Vgat neurons enhanced scratching behaviors. The overall effect of lidocaine therefore could be explained by a prominent role of inhibition of the Vglut2 neurons. Furthermore, activating Vgat neurons attenuated scratching, mimicking the effects of global PAG activation. It is tempting to speculate that these results can be explained by a microcircuit in the vlPAG, where Vgat neurons exert inhibitory control over Vglut neurons, which in turn provide descending projections that can enhance itch behaviors. These results clearly highlight the differences in pharmacological, global and cell-type specific manipulations, and as such the behavioral outcomes using global pharmacological, electrophysiological, or other types of stimulation or inhibition need to be carefully interpreted.

Global chemogenetic inhibition or activation of vlPAG neurons has the same effect on both pain[1] and itch behaviors. Namely, global inhibition of the vlPAG potentiates both pain and itch while global activation of vlPAG neurons attenuates both pain and itch. These results are consistent with the hypothesis that the overall function of PAG output is to inhibit both pain and itch transmission. However, the results of experiments in which we selectively modulated activity of either GABAergic or glutamatergic neurons suggest that the mechanisms by which the PAG modulates pain and itch transmission are more complex, as presented schematically in Figure 10. Pain and itch are known to be processed distinctively at the spinal level. Using cell type specific chemogenetic manipulations, we found that activating GABAergic or inhibiting glutamatergic neurons in vlPAG causes suppression of itch and potentiation of pain [1], whereas inhibiting GABAergic or activating glutamatergic neurons causes potentiation of itch and suppression of pain behaviors. Our findings demonstrate that also at the midbrain level, vlPAG GABAergic neurons and glutamatergic neurons process both pain and itch signals inversely.

Thus, although pain and itch are processed and transmitted by similar neuroanatomical substrates at peripheral, spinal and supraspinal sites, they are discriminated and processed distinctly at the cellular level.

We found that manipulating the activity of PAG neurons during chloroquine-evoked scratching leads to alterations in spinal cord activity, as we assessed using cFos expression studies, suggesting that vlPAG neurons modulate spinal pruritic processing. The PAG forms strong connections with the amygdala, the habenula, several thalamic and hypothalamic nuclei, the RVM and the locus coeruleus [2, 3, 4, 5, 6, 7, 8, 9]. We hypothesize based on prior work[10] that PAG neurons might be modulating spinal cord activity via projections to RVM. In future studies, it will also be of great interest to determine which of these projection targets, or others, might mediate the differential modulation of pain and itch from glutamatergic and GABAergic projections from the vlPAG. The balance of activity in the vlPAG might lead to differential modulation of the activity in downstream projection targets and contribute to the inverse control of pain and itch processing in the spinal cord.

Supplementary References

1. Samineni VK*, et al.* Divergent Modulation of Nociception by Glutamatergic and GABAergic Neuronal Subpopulations in the Periaqueductal Gray. *eNeuro* **4**, (2017).

2. Behbehani MM. Functional characteristics of the midbrain periaqueductal gray. *Prog Neurobiol* **46**, 575-605 (1995).

3. Heinricher MM, Tavares I, Leith JL, Lumb BM. Descending control of nociception: Specificity, recruitment and plasticity. *Brain Res Rev* **60**, 214-225 (2009).

4. Mantyh PW. Connections of midbrain periaqueductal gray in the monkey. I. Ascending efferent projections. *J Neurophysiol* **49**, 567-581 (1983).

5. Mantyh PW. Connections of midbrain periaqueductal gray in the monkey. II. Descending efferent projections. *J Neurophysiol* **49**, 582-594 (1983).

6. Reichling DB, Basbaum AI. Collateralization of periaqueductal gray neurons to forebrain or diencephalon and to the medullary nucleus raphe magnus in the rat. *Neuroscience* **42**, 183-200 (1991).

7. Rizvi TA, Ennis M, Shipley MT. Reciprocal connections between the medial preoptic area and the midbrain periaqueductal gray in rat: a WGA-HRP and PHA-L study. *J Comp Neurol* **315**, 1-15 (1992).

8. Hamilton BL, Skultety FM. Efferent connections of the periaqueductal gray matter in the cat. *J Comp Neurol* **139**, 105-114 (1970).

9. Mason P. Deconstructing endogenous pain modulations. *J Neurophysiol* **94**, 1659-1663 (2005).

10. Gao ZR*, et al.* Tac1-Expressing Neurons in the Periaqueductal Gray Facilitate the Itch-Scratching Cycle via Descending Regulation. *Neuron* **101**, 45-+ (2019).
